# Research on Chinese Speech Emotion Recognition Based on Deep Neural Network and Acoustic Features

**DOI:** 10.3390/s22134744

**Published:** 2022-06-23

**Authors:** Ming-Che Lee, Sheng-Cheng Yeh, Jia-Wei Chang, Zhen-Yi Chen

**Affiliations:** 1 Department of Computer and Communication Engineering, Ming Chuan University, Taoyuan 333, Taiwan; leemc@mail.mcu.edu.tw (M.-C.L.); peteryeh@mail.mcu.edu.tw (S.-C.Y.); s742238@gmail.com (Z.-Y.C.); 2 Department of Computer Science and Information Engineering, National Taichung University of Science and Technology, Taichung City 404, Taiwan

**Keywords:** emotion recognition, deep neural network, acoustic features

## Abstract

In recent years, the use of Artificial Intelligence for emotion recognition has attracted much attention. The industrial applicability of emotion recognition is quite comprehensive and has good development potential. This research uses voice emotion recognition technology to apply it to Chinese speech emotion recognition. The main purpose of this research is to transform gradually popularized smart home voice assistants or AI system service robots from a touch-sensitive interface to a voice operation. This research proposed a specifically designed Deep Neural Network (DNN) model to develop a Chinese speech emotion recognition system. In this research, 29 acoustic characteristics in acoustic theory are used as the training attributes of the proposed model. This research also proposes a variety of audio adjustment methods to amplify datasets and enhance training accuracy, including waveform adjustment, pitch adjustment, and pre-emphasize. This study achieved an average emotion recognition accuracy of 88.9% in the CASIA Chinese sentiment corpus. The results show that the deep learning model and audio adjustment method proposed in this study can effectively identify the emotions of Chinese short sentences and can be applied to Chinese voice assistants or integrated with other dialogue applications.

## 1. Introduction

Language is the main way for people to communicate. In addition to the message meaning contained in language, it also contains the transmission of emotions. Through emotions, tone, and other messages; even if the other party does not understand the meaning of the message in the language, one can still feel the speaker’s emotions in words. In recent years, the use of artificial intelligence and deep learning for emotion recognition has attracted much attention. The industrial applicability of emotion recognition is quite comprehensive and has good development potential. In various applications in daily life, human–computer interaction has gradually been replaced by voice operations and dialogues from touch-sensitive interfaces. Speech recognition is widely used in transportation, catering, customer service systems, personal health care, and leisure entertainment [1,2,3,4,5]. In recent years, Automatic Speech Recognition (ASR) technology has matured and has been able to accurately recognize speech and convert it into text [6,7,8]. However, in addition to the meaning of language itself that can convey information between dialogues, the emotions accompanying the dialogue are also important information. Since emotions are full of information, Automatic Emotional Speech Recognition (AESR) technology will be the focus of the next generation of speech technology. In recent years, the use of deep-learning-related technologies to recognize speech emotions has increased rapidly. Li et al. [9] used a hybrid deep neural network combined with a Hidden Markov Chain to construct a speech recognition model, achieving significant effects in the EMO-DB dataset. In the research of Mao et al. [10] and Zhang et al. [11], it was verified that a convolutional neural network can effectively learn the emotional features in speech. In Umamaheswari, J., and Akila, A. [12], a Pattern Recognition Neural Network (PRNN) combined with a KNN algorithm was first tried, and the results were better than the traditional HMM and GMM algorithms. Mustaqeem and Soonil Kwon [13] proposed a deep stride strategy to construct spectrogram feature maps and achieved good identification performance in the well-known IEMOCAP and RAVDESS datasets. In 2021, Li et al. [14] proposed a bi-directional LSTM model combined with a self-attention mechanism to recognize speech emotions, which achieved remarkable performance in well-known and corpora IEMOCAP and EMO-DB. The proposed model achieved the highest recognition accuracy in the recent period in the recognition of ‘Happiness’ and ‘Anger’ emotions. Until now, most of AESR’s research has mainly focused on English or European languages [15,16], and research on the recognition of Chinese speech emotions by deep neural networks is relatively rare. This research proposes a Chinese speech emotion recognition model based on deep networks and combines audio and elevation adjustments to explore the effectiveness of audio features in deep networks.

The remainder of this paper is as follows: Section 2 discusses system-related technologies and studies; Section 3 proposes research frameworks and methods; Section 4 is the experimental design and results analysis; and the final section provides the conclusions.

## 2. Related Techniques and Literature Review

### 2.1. Acoustic Features

The extraction and selection of acoustic features is an important part of speech recognition. In sound analysis, short-term analysis is usually the main method. The sound is cut into several frames and then analyzed according to the signal in each frame. Three main sound characteristics can be observed, as follows:

Volume: in terms of the amplitude of the sound, the greater the amplitude, the greater the volume of the sound waveform.

Pitch: this expresses the sound level by frequency; the higher the basic frequency of the sound, the higher the pitch.

Timbre: Timbre represents the content of the sound, which can be represented by the change in each waveform in a basic cycle. Different timbres represent different audio content.

Recently, there has been extensive research on specific features related to emotions in speech and audio. In Schuller et al. [17], short-term analysis was used to define 6373 feature sets. In addition, Eyben et al. [18] proposed a set of minimalistic features in the Geneva Minimalistic Acoustic Parameter Set (GeMAPS), consisting of 62 features. The following describes the sound characteristics used in this study.

#### 2.1.1. Spectral Centroid

**Figure 1:**
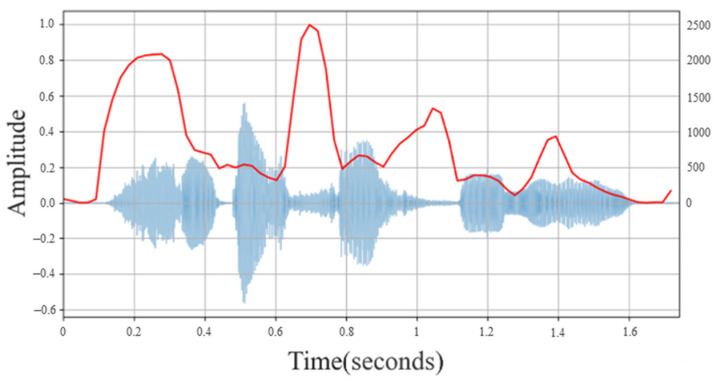
Spectral Centroid Waveform.

The spectral centroid [19] is an important parameter describing the characteristics of timbre. It is used to describe the frequency centroid of a sound signal in a spectrogram and can express the frequency distribution trend of each frame. The spectral centroid of each frame is drawn into a waveform graph. Figure 1 is a representation of the spectral energy distribution. Through the distribution of the overall average energy within a certain frequency range, it can be explained that the average value of the signal component frequency is biased towards high or low frequencies. In the physical sense, the spectral centroid can describe the brightness of a sound. When the sound is dark and deep, the frequency is more low frequency, and the spectral centroid is relatively low; when the sound is bright and brisk, usually concentrating on high frequencies, the spectral centroid is relatively high. The formula is shown in Equation (1), dividing the frequency spectrum into *N* frequency bands, where x(n) is the nth energy intensity (Magnitude) corresponding to the frequency *f*(n).

(1) $$SC=\frac{\sum_{n=1}^{N}f\left(n\right)x\left(n\right)}{\sum_{n=1}^{N}x\left(n\right)}$$

#### 2.1.2. Spectral Flatness

Spectral flatness [20] indicates the average degree of energy distribution among audio frequency bands. Divide the spectrum into *N* frequency bands, where x(n) represents the total energy intensity of the nth frequency band, and then calculate the geometric average and arithmetic average of x(n), respectively, and express the rate of change as the ratio, as in Equation (2). Since the arithmetic average is greater than the geometric average, the calculation result is between 0~1. When the energy distribution of each frequency band is average, the ratio will approach 1; otherwise, it will approach 0.

(2) $$SF=\frac{\sqrt[{N}]{\prod_{n=1}^{N}x\left(n\right)}}{\frac{\sum_{n=1}^{N}x\left(n\right)}{N}}$$

#### 2.1.3. Spectral Contrast

The concept of spectral contrast is that each frame in the spectrogram is divided into sub-bands. Energy contrast is obtained by calculating the average energy in the spectral peaks and spectral valleys in the sub-bands (i.e., Peak Energy and Valley Energy) [21]. High contrast represents clear sound signals and narrow-band signals, while low contrast represents noise.

#### 2.1.4. Spectral Roll-Off

Spectral roll-off refers to the center frequency of the amplitude distribution below a specified percentage [22]. This feature is usually used to distinguish between voiced speech and clear speech. The energy of clear speech is mostly concentrated in the high-frequency range.

#### 2.1.5. Chroma Feature

**Figure 2:**
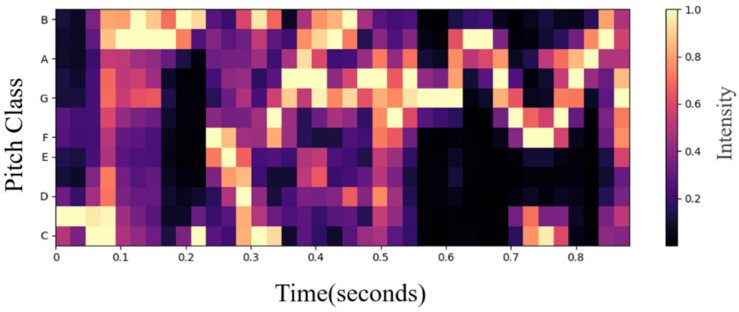
Chromagram obtained from a voice recording.

Chroma features describe, as a collective term, Chroma Vectors and Chromagrams. The chromaticity vector contains 12 elements, which are *C*, *C#*, *D*, *D#*, *E*, *F*, *F#*, *G*, *G#*, *A*, *A#*, and *B*. These elements represent the energy of the 12 sound levels in a period (such as one frame). The energy of the same sound level for different octaves is accumulated, and the chromaticity map is the chromaticity vector sequence. The twelve equal temperament is a method of using equations for musical rhythm [23]. The chroma vector is composed of a vector of 12 element features, used to represent the energy in each scale in the signal. The visualized Chromagram is shown below Figure 2:

#### 2.1.6. Zero-Crossing Rate (ZCR)

ZCR is the number of times the audio passes through the zero point in each frame [24]. The equation is as (3), where *s* is a signal with length *T*, the function π{A} is 1 while parameter A is true; otherwise, it is 0. This feature can show frequency characteristics and has been widely used in the field of speech recognition and music information retrieval. Usually, the ZCR of noise and air noise is larger than that of normal sound.

(3) $$\mathrm{ZCR}=\frac{1}{T-1}\overset{T-1}{\underset{t=1}{\sum }}\pi \left\{s_{t}s_{t-1}<0\right\}$$

#### 2.1.7. Root Mean Square Energy (RMSE)

RMSE calculates the root mean square value of each frame. The equation is as (4), *N* is the total number of frames and *y*(*n*) is the audio information of the *n*-th frame.

(4) $$\mathrm{RMSE}=\sqrt{\frac{1}{N}\overset{N}{\underset{n=1}{\sum }}{\left|y\left(n\right)\right|}^{2}}$$

#### 2.1.8. Mel Frequency Cepstral Coefficient (MFCC)

In speech recognition, Mel Frequency Cepstral Coefficient (MFCC) is one of the most commonly used voice features. Fletcher and Munson [25] pointed out that the human ear has different sensitivity to sound waves of different frequencies and different loudness. When two sound waves with different loudness and the same frequency act on the human ear, the high-loudness audio will affect the human ear’s perception of the lower loudness audio, making the low-loudness signal difficult to notice. This phenomenon is called the masking effect. The sound with lower frequency has a greater distance of wave transmission than the sound with higher frequency, so the bass can easily cover the treble. The process of MFCC feature extraction in this study is as follows:

(1) Pre-emphasis: The signal is pre-emphasized, and the voice signal is passed through a high-pass filter, as in Equation (5), where y(n) is the output signal, x(t) is the original signal, and the value of α is usually between 0.9 and 1.0; in this study, the default was 0.97. Pre-emphasis will boost the high-frequency part and flatten the spectrum of the audio, maintaining it in the entire frequency band from low to high frequencies, using the same signal-to-noise ratio to obtain the spectrum.
  (5) y(n)=x(t)−αx(t−1)
(2) Frame blocking and Hamming: Frame blocking collects N sampling points into a frame and the value of N is set to 512. Subsequently, each frame is multiplied by a Hamming window to increase the continuity at the left and right ends of the frame. Assuming that the framed signal is S(n), n=0,1…,N−1, N is the frame size and the windowed signal is as in Equation (6), and the Hamming window is calculated as in Equation (7), where a is set as 0.46 by default. After using the Hamming window, each frame is fast Fourier transformed to obtain the energy distribution in the spectrum, and the logarithmic energy and signal characteristics are obtained by 20 triangular bandpass filters.
  (6) $${S^{\prime }}^{\left(n\right)}=S\left(n\right)-W\left(n\right)$$
  (7) $$W\left(n,a\right)=\left(1-a\right)-a\times \cos\left[\frac{2\pi n}{N-1}\right],0\leq n\leq N-1$$

### 2.2. Speech Representation and Emotion Recognition

After the recent rapid development of artificial intelligence technologies, such as machine learning and deep learning, affective computing began to appear in various applications, such as robot dialogue and medical care. Affective computing infers the user’s emotions and responds by sensing and understanding the differences in human faces, gestures, and speech in different states. In this field, emotion recognition with pure speech is the most challenging and the most widely used technology, and the development of this field is highly dependent on the construction of emotional speech datasets. The construction of the emotional speech corpus can be roughly divided into two categories.

The first type is guided recording, which is mostly recorded in a laboratory or a recording studio. It is recorded through high-quality microphones and guided by linguistic experts. These types of data can generate an emotional corpus with high emotional expression and diversity. Representative sentiment corpora include: Emo-DB [26], recorded by the Technical University of Berlin, Germany, with 10 actors (5 males and 5 females), performing 10 German voices, containing a total of 800 sentences. IEMOCAP [27], recorded by the University of Southern California, including 10 actors performing a session, a total of 5 sessions, and each utterance is assessed by at least three experts. CASIA [28], a Chinese sentiment corpus, recorded by the Institute of Automation of the Chinese Academy of Sciences, where the voice data were recorded by two men and two women with 500 different texts.

Another corpus type is non-lab recording. The difference between this type of corpus and guided recording is that it is made up of spontaneous emotional expression sentences of natural scenes, for example, living environment, theatrical performance paragraphs, etc. This type of corpus is a relatively new corpus, such as: NNIME [29], the NTHU-NTUA Chinese Interactive Emotion Corpus, is a performing-arts-type corpus. It combines speech, drama, body language, and scene design. CHEAVD [30], CASIA Chinese Natural Emotional Audio–Visual Database. The corpus extracts 140 min emotional clips from movies, TV dramas, and talk shows. The actors include a total of 238 people, from children to the elderly, and they are annotated by 4 native Chinese speakers. 

This study adopts the public version of the CASIA Chinese sentiment corpus. The emotional sounds are divided into six categories: ‘*Happiness*’, ‘*Sadness*’, ‘*Angry*’, ‘*Fright*’, ‘*Calm*’, and ‘*Fear*’. Compared to the underlying emotion–cognitive dimensions, such as James Russell Arousal-Valence four-quadrant model [31], the six emotions belonging to quadrants I, III, II, I, IV, and II, respectively. 

In recent years, deep learning has made great progress in speech representation. Baevski and Schneider et al. [32,33] proposed a wav2vec model, which is an unsupervised speech recognition system. The framework uses only 10 min of transcribed speech data to support automatic speech recognition models. In 2021, Hsu et al. proposed a speech pre-training model [34] that surpasses wav2vec 2.0. The authors in [34] pointed out that there are several problems in the unsupervised learning of speech, including that there are many pronunciation units in speech, the lengths of pronunciation units are different, and the units of speech have no fixed segmentation, etc. For these problems, the idea of [34] is to label the predicted values in a clustering manner, and then mask the labels as unsupervised learning targets. Meanwhile, researchers at Microsoft Research Asia proposed a method called UniSpeech [35]. UniSpeech is able to leverage both supervised and unsupervised data to learn a unified contextual representation. The model includes a feature extraction network based on a convolutional neural network, and a context network of a Transformer model and a feature quantization module for learning discrete vectors. In a specific setting, UniSpeech is significantly better than supervised transfer learning. Further, in 2021, researchers from Microsoft Research Asia and Microsoft Azure Speech Group proposed a general speech pre-training model, WavLM [36], which achieved state-of-the-art performance on multiple speech datasets. 

Although voice representation approaches can effectively provide text or vector representation at the coding level, they cannot judge the user’s emotions at the application level. Speech emotion recognition requires a speech emotion database for training. The public emotion corpora commonly used in recent studies are the German Berlin Database of Speech Emotion [26], FAU Aibo [37], and the Ryerson Audio-Visual Database of Emotional Speech and Song (RAVDESS) [38]. A typical machine learning speech emotion recognition system includes speech input, feature extraction, classification models, and emotional output recognition. Commonly used classification models include SVM [39], HMM [40], and Gaussian Mixture Model (GMM) [41].

Lin and Wei [28] used SVM and HMM classification methods to identify different categories of emotions, such as angry, happy, sad, surprised, and calm. In total, 39 candidate features were extracted and Sequential Forward Selection (SFS) was used. The method finds the best feature subset and the final average recognition accuracy of the HMM classifier is 99.5%; the SVM classifier is 88.9%. Lim et al. [42] first performed Short-Time Fourier Transform (STFT) on the voice data into a spectrogram, putting it in series with a Convolutional Neural Network (CNN) and Recurrent Neural Network (RNN) model for speech emotion recognition, with emotions including: ‘*Angry*’, ‘*Happy*’, ‘*Sad*’, ‘*Calm*’, ‘*Fearful*’, ‘*Disgust*’, and ‘*Bored*’. Its model is to combine four-layer CNN with a long short-term memory network (long short-term memory, LSTM), and the final emotion recognition accuracy rate is 88%.

## 3. System Architecture and Research Method

### 3.1. System Architecture

**Figure 3:**
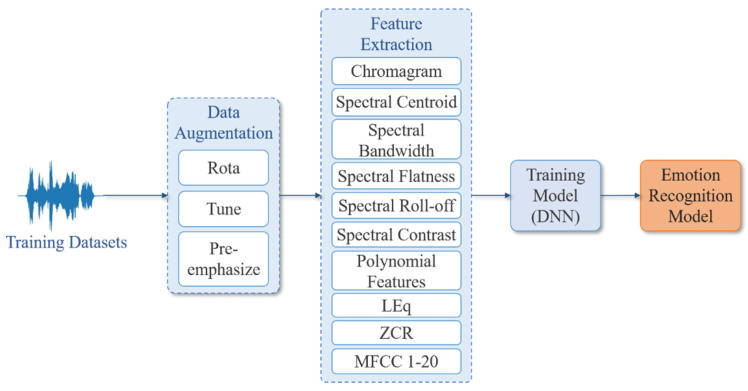
Training flowchart of the proposed emotion recognition model.

The main concept of this research is to extract the acoustic features of Chinese speech for sentiment analysis and classification. Using the method of sound rotation and sound frequency modulation, amplify the training samples and extract 29 acoustic features from the sound signal, and input the DNN model proposed in this research for training. The system architecture is shown in Figure 3. Due to the shortcomings of the Chinese language and corpus, this study designed two algorithms for augmenting data. This research first divides the collected voice emotion dataset into training data and test data and increases the amount of voice emotion data through two voice data extension methods. As such, 29 voice features are extracted, respectively: “*Chroma Feature*”, “*Spectral Centroid*”, “*Spectral Bandwidth*”, “*Spectral Flatness*”, “*Spectral Roll*-*off*”, “*Spectral Contrast*”, “*Polynomial Features*”, “*RMSE*”, “*ZCR*”, and “*MFCC* 1-20”. Finally, the classification result is obtained through the proposed DNN model.

### 3.2. The Proposed DNN Model

**Figure 4:**
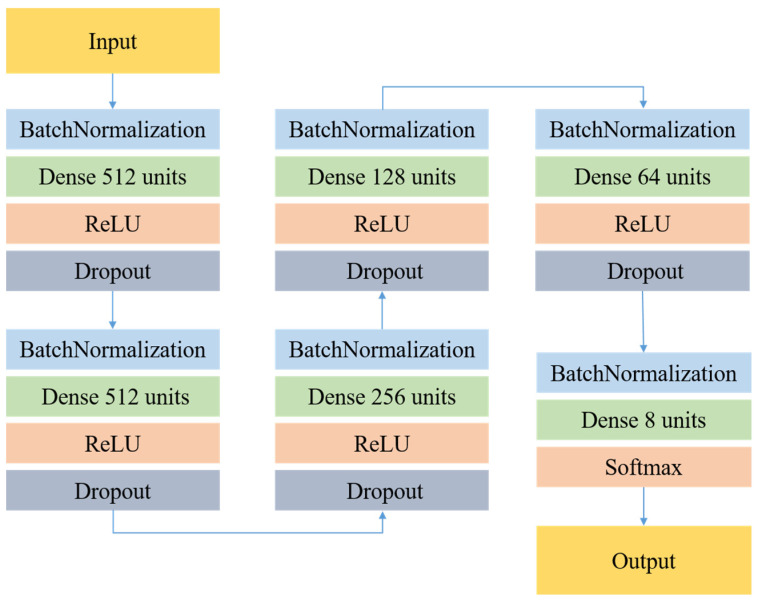
The proposed Deep Neural Network Model.

The following figure is the DNN model proposed in this research. This DNN model uses ReLU function and Dropout in the hidden layer, and the output layer uses the Softmax function. As shown in Figure 4, 29 dimensional features are used as input, and the model contains 5 hidden layers with 512, 512, 256, 128, 64, and 8 neurons, respectively. In this model, a decreasing network architecture can effectively generalize acoustic features to a single emotional label. The final Softmax layer can produce the probability output of a single sentiment label. In the prediction phase, this study will use the category with the highest probability value as the prediction output. The emotion training dataset in this study uses CASIA Chinese Emotion Corpus, which is recorded by 4 professional speakers (2 males and 2 females) in Chinese accents with various emotions. The CASIA sentiment corpus has a total of 9600 speeches, including 6 emotions and 300 sentences from the same text and 100 sentences from different texts.

### 3.3. Data Augmentation

Because of the scarcity of Chinese speech emotion data, this study proposes a voice augmentation approach to obtain more sufficient data. There are four main data expansion methods in the audio field, namely: sound rotating, pitch adjustment (tuning), clipping, and noising. The conversions will not change the label of the original data but increase the variability of the data in the original category.

#### 3.3.1. Waveform Adjustment

**Figure 5:**
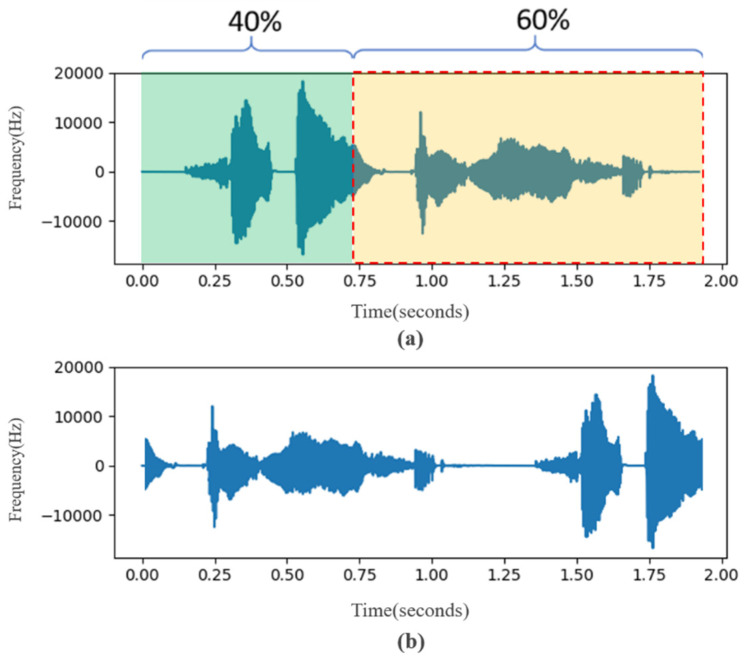
(**a**) Original waveform (**b**) waveform after 60% rotation.

**Figure 6:**
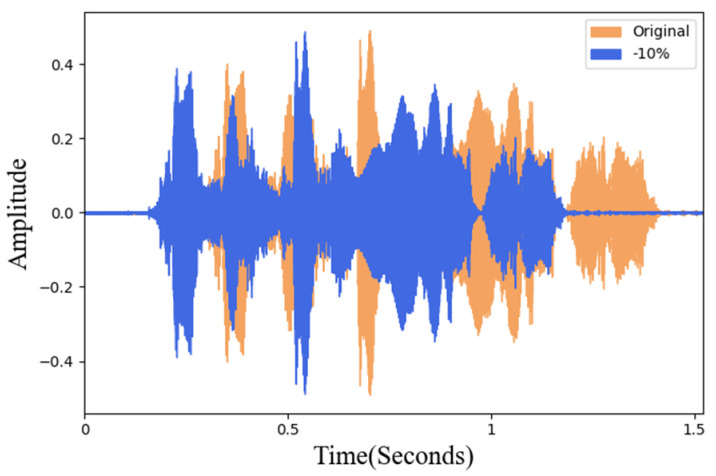
Waveform of frequency adjustment and original data.

In this study, different degrees of sound rotation and two pitch adjustments were tested. In order to avoid the difference between the new data and the original data being too small, the sound rotation was taken as a unit of 10% and 10~90% was performed, respectively. Figure 5a is the waveform of the original data and Figure 5b is the waveform after 60% rotation. In sound frequency modulation adjustment, this research uses 5% as a unit to adjust the frequency amplitude. To maintain the natural intelligibility of the adjusted data, after the actual listening test, the maximum value can be adjusted to 30%. Therefore, frequency modulation processing of plus or minus 30% is carried out, respectively. Figure 6 is a waveform diagram of the comparison of the 10% frequency reduction in the original data. In Figure 6, the orange sound wave represents the original data and the blue is the data processed by frequency modulation.

#### 3.3.2. Pitch Adjustment

**Figure 7:**
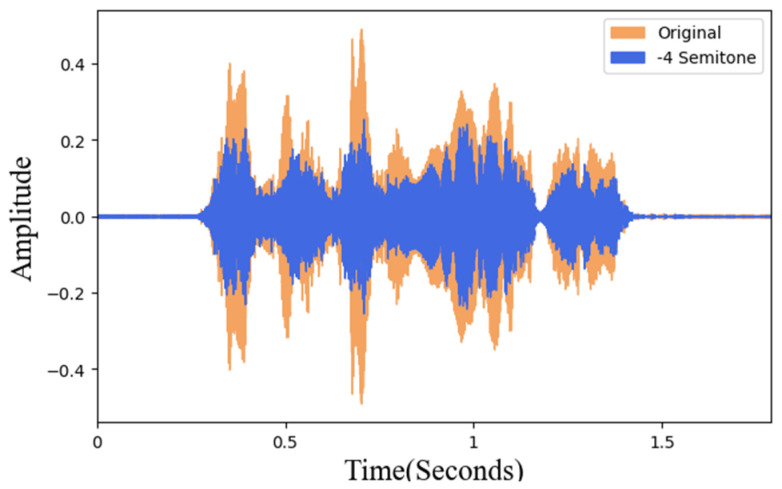
Pitch adjustment and original data waveform.

In this study, the pitch is adjusted in a semitone unit. To maintain the natural intelligibility of the adjusted data, after the actual listening test, the maximum value can be adjusted to 6 semitones. Therefore, pitch processing of plus and minus 6 semitones is performed, respectively. Figure 7 shows the 4 semitones for the voice data waveform comparison diagram with original data. The orange sound wave is the original data and the blue is the pitch-adjusted data. The overall time and frequency remain unchanged and the purpose of changing the pitch can also be achieved.

#### 3.3.3. Pre-Emphasize

**Figure 8:**
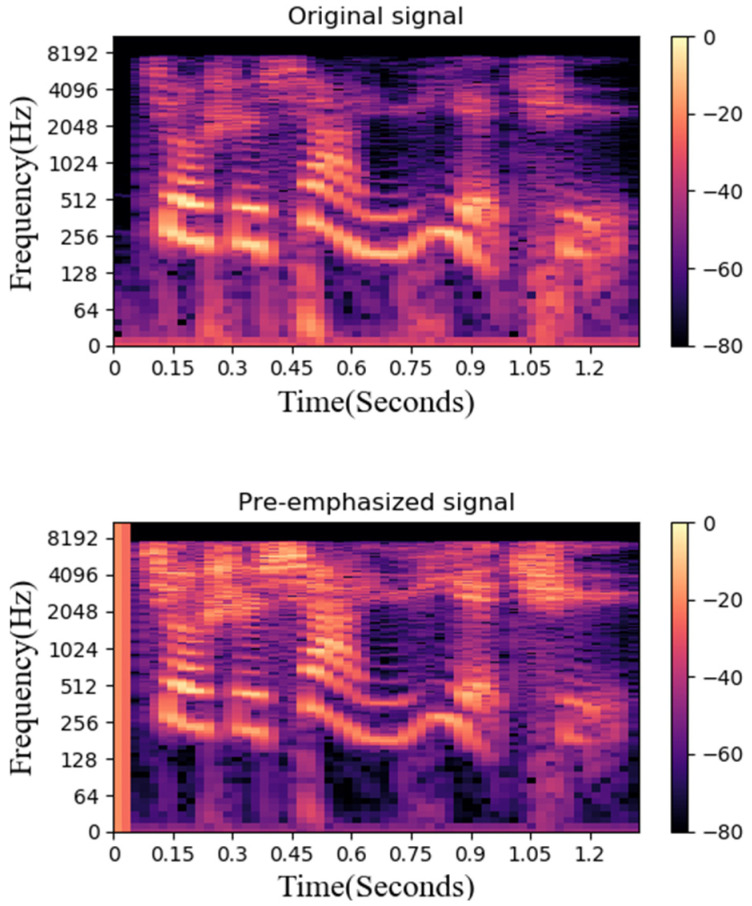
Original data and spectrogram after pre-emphasize.

This study uses Mel Scale to pre-emphasize the original speech signal. The Mel Scale is the non-linear characteristic of the human ear frequency, which can be approximated by a mathematical conversion of *Hz*. This study uses a set of twenty triangular bandpass filters to obtain log energy and obtains representative coefficients of different frequency bands through cosine conversion. Figure 8 is a comparison between the pre-emphasis and the original data. It can be found that the sound characteristics are more obvious after processing.

## 4. Experimental Results and Discussion

### 4.1. Experimental Environment

The emotional sounds are divided into six categories: ‘*Happiness*’, ‘*Sadness*’, ‘*Angry*’, ‘*Fright*’, ‘*Calm*’, and ‘*Fear*’. The experimental voice was recorded in a pure environment without background noise. The signal-to-noise ratio is about 35 dB. The voice files are stored in WAV format with a sampling rate of 16,000 and a sampling resolution of 16 bit. This study adopts the public version of the CASIA Chinese sentiment corpus. Each sentiment is 200 samples containing 50 sentences. The OS of the experimental environment is Windows 10; DNN is built using TensorFlow, with NVIDIA GeForce GTX 1050 Ti GPU, and memory of 4 Gbytes. DNN parameter settings: 300 epochs, 100 batch size, 25% of the verification data, and 20% testing data from the original data.

### 4.2. Experimental Results of the Original Method

**Figure 9:**
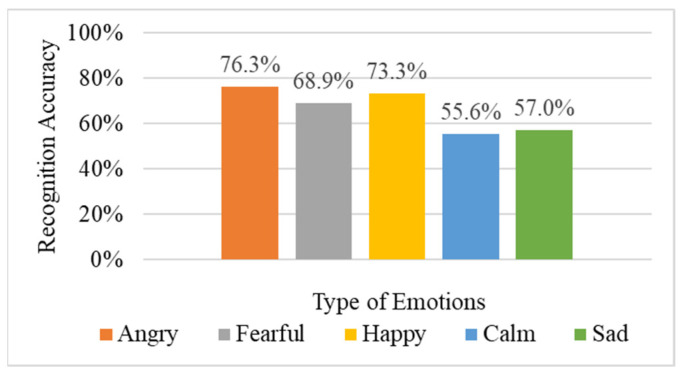
Results of the original method.

In this experiment, 29 sound features were extracted from the training data and input into the DNN for training without any sound data extension. The 29 acoustic features include spectral centroid, spectral bandwidth, equivalent sound level, spectral roll-off, ZCR summarized by GeMAPS [18], plus four common sound features: spectral flatness, chroma feature, spectral contrast, polynomial features, and the first 20 numerical outputs of MFCC, which were mentioned in Section 2.1. Figure 9 shows the average recognition accuracy of the emotion recognition results. In the original method, ‘*Angry*’ and ‘*Happy*’ can be distinguished more clearly, while the recognition accuracy of ‘*Calm*’ and ‘*Sad*’ is relatively low. These two emotions are relatively smooth and not obvious. The overall average recognition rate is 66.2%.

### 4.3. Experimental Results of the Pre-Emphasize

**Figure 10:**
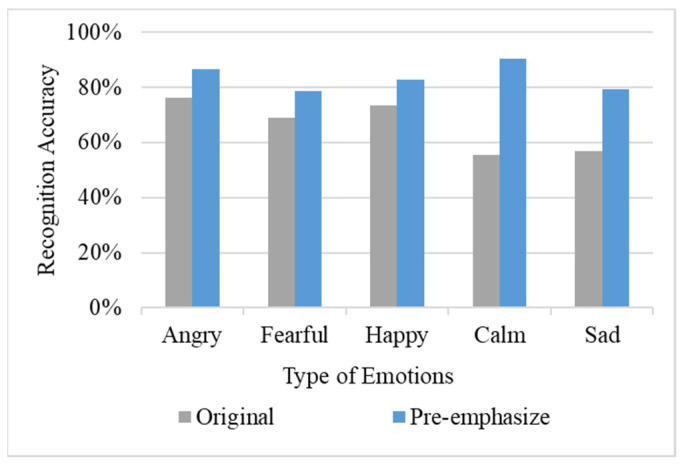
Results of the pre-emphasize procedure.

Subsequently, this study used the pre-emphasis method to expand the data size to 2400; the results are shown in Figure 10. The results show that the pre-emphasis method has a significant increase in the recognition accuracy of each emotion and the average recognition accuracy is increased to 83.6%. Table 1 shows the test results of pre-emphasized data, especially the recognition accuracy of ‘*Calm*’ and ‘*Sad*’, which rose to 80%. The recognition errors caused by unobvious features were improved by the pre-emphasis procedure.

### 4.4. Rotating

In this experiment, we rotated the original data according to different degrees, and the data size after extension was 2400. Table 2 shows the testing results with different rotation degrees. The experimental results show that due to the increase in the amount of data, the accuracy of emotion recognition has increased significantly. The improvement in accuracy is most obvious with an average recognition rate of 84.7% at the 40% level. The difference between the new data generated by sound rotation and the original data will increase the diversity of the overall training data. Among them, the recognition rates of ‘*Happy*’ and ‘*Calm*’ are obvious, rising to about 88%. The average recognition accuracy of ‘*Angry*’ is 1.2-times that of the original data, while ‘*Sad*’ maintains the original accuracy.

### 4.5. Pitch Adjustment Analysis

#### 4.5.1. Sound Frequency Adjustment

In the sound frequency adjustment, we adjusted the frequency amplitude in units of 5%. To maintain the natural intelligibility of the adjusted data, the maximum value can be adjusted to 30% after the actual listening test. Table 3 is the result of training the DNN model and then testing after adding different frequency modulation data. From the overall observation, it can be found that the method of reducing the frequency has a more significant improvement in the recognition accuracy compared to the method of increasing the frequency. The reason for this result is that increasing the frequency will promote the compression of the sound signal, resulting in distortion of the voice data. Therefore, the higher the frequency increases, the more the recognition accuracy decreases. This method has the most significant effect at the −10% level. Its average recognition accuracy rate is 86.5%, which is the same as voice rotation. The recognition rates of ‘*Happy*’ and ‘*Calm*’ were significantly improved, and the recognition accuracy of ‘*Sad*’ was also improved, which is generally better than that of voice rotation.

#### 4.5.2. Pitch Adjustment

This experiment is adjusted based on rising or falling one semitone. The results are shown in Table 4. In terms of results, the average recognition accuracy of the sound frequency modulation method has better results than the pitch adjustment method. Therefore, the sound adjustment method will be used for the pitch adjustment. The next experiment will use the −10% extension in the sound frequency modulation method.

### 4.6. Comprehensive Adjustment

As mentioned earlier, the experiment found that the best choice of sound rotation level is 40%, with FM-10% and pre-emphasis for different combinations. The data volume after extension is also 2400 for the experiment. Table 5 shows the experimental results after adjusting the data. The best model is rotating 40% with FM-10%. Compared with the original data, the recognition rate of ‘*Angry*’ was greatly increased to 93.3%; the recognition accuracy of ‘*Happy*’ also increased by 24.5% and it rose to 97.8%. Among them, ‘*Calm*’ has the largest growth rate. The accuracy of significant recognition increased by 37.7%; ‘*Sad*’ increased by 20.8% compared to the original data. The average recognition rate increased by 22.7% and the average recognition rate of the best model with 40% rotation and FM-10% was 88.9%.

Table 6 is a confusion matrix with an average recognition rate of 40% rotation and FM-10% model test. The ‘*Happy*’, ‘*Angry*’, and ‘*Calm*’ results are all excellent, and the recognition accuracy can reach more than 90%, but ‘*Fearful*’ and ‘*Sad*’ are found to be less recognized. The reason is that these two primitive emotions have a higher chance of being confused, but they were originally identified as ‘*Fearful*’, and vice versa.

This study also compares the training time, training recognition, verification recognition, and test recognition accuracy with K-nearest-neighbors and GoogLeNet [43]. The results are shown in Table 7, Table 8, Table 9 and Table 10. In this experiment, audio spectrograms are generated by fast Fourier transform via original audios and as input to the GoogLeNet model. The results show that GoogLeNet has the longest training time, and the proposed DNN has the highest emotion recognition accuracy on average.

## 5. Conclusions and Future Work

In this study, two sound data extension methods were used to extend the data and increase the variability of the data in the original type of data, thereby improving the accuracy of identification. In the experiments, we applied the extension methods to all samples, including training, validation, and testing data. In sound frequency modulation, the impact of different frequency data on the recognition rate was tested through the extension method, and it was found that the conversion to high frequency may produce distortion in the voice data. The experiment found that the result of 10% frequency adjustment is the best, and more voice data of different frequencies can be obtained. In sound rotation, experiments were conducted based on the difference in the degree of rotation, and it was found that the effect of 40% rotation was the most prominent. Therefore, it is judged that this degree of rotation is quite different from the original data, which promotes the variability of the training data. After the final comprehensive adjustment, the optimal sound rotation degree and the sound frequency modulation degree are combined into training data and 29 sound features are extracted and input into the specially designed DNN for training in this study. The final average recognition accuracy of speech emotion is up to 88.9%.

In future studies, several parts can be improved to increase the emotion recognition rate. The first is to increase the amount of Chinese speech and emotion data. The second part is to increase the gender recognition ability. If the data can be distinguished from gender in the pre-training stage, or the gender label can be added, it is expected to effectively improve the recognition accuracy. In addition, future research will try to use different types of deep network models, such as Attention Mechanism and Transformer models, combining with acoustic features for training and performance evaluation.

## Figures and Tables

**Table 1:** Comparison of original method and pre-emphasized results.

Model	Angry	Fearful	Happy	Calm	Sad	Average
**Original**	76.3%	68.9%	73.3%	55.6%	57.0%	66.2%
**Pre-emphasize**	86.7%	78.5%	83.0%	90.4%	79.3%	83.6%

**Table 2:** Comparison of original method and rotating results.

Model	Angry	Fearful	Happy	Calm	Sad	Average
**Original**	76.3%	68.9%	73.3%	55.6%	57.0%	66.2%
**10%**	83.7%	74.8%	91.9%	86.7%	80.7%	83.6%
**20%**	83.7%	77.8%	88.9%	87.4%	83.0%	84.1%
**30%**	83.0%	68.9%	88.1%	87.4%	84.4%	82.4%
**40%**	87.4%	80.0%	91.1%	88.9%	76.3%	84.7%
**50%**	80.7%	73.3%	89.6%	85.9%	78.5%	81.6%
**60%**	83.7%	75.6%	91.1%	87.4%	80.0%	83.6%
**70%**	82.2%	69.6%	88.1%	85.2%	86.7%	82.4%
**80%**	83.0%	73.3%	89.6%	85.2%	83.7%	83.0%
**90%**	82.2%	72.6%	91.1%	87.4%	79.3%	82.5%

**Table 3:** Comparison of original method and sound frequency adjustment.

Model	Angry	Fearful	Happy	Calm	Sad	Average
**Original**	76.3%	68.9%	73.3%	55.6%	57.0%	66.2%
**−30%**	84.4%	77.8%	89.6%	93.3%	78.5%	84.7%
**−25%**	89.6%	77.0%	91.9%	93.3%	79.3%	86.2%
**−20%**	83.7%	76.3%	94.1%	91.9%	82.2%	85.6%
**−15%**	88.1%	77.0%	92.6%	90.4%	82.2%	86.1%
**−10%**	85.9%	78.5%	93.3%	91.1%	83.7%	86.5%
**−5%**	85.2%	72.6%	94.8%	88.9%	81.5%	84.6%
**5%**	85.2%	71.9%	85.9%	90.4%	82.2%	83.1%
**10%**	85.9%	77.0%	89.6%	92.6%	80.7%	85.2%
**15%**	86.7%	80.7%	88.9%	87.4%	77.8%	84.3%
**20%**	80.7%	74.8%	90.4%	86.7%	79.3%	82.4%
**25%**	82.2%	76.3%	88.9%	85.2%	84.4%	83.4%
**30%**	77.2%	71.1%	90.0%	83.3%	83.9%	81.1%

**Table 4:** Comparison of original method and sound pitch adjustment.

Model	Angry	Fearful	Happy	Calm	Sad	Average
**Original**	76.3%	68.9%	73.3%	55.6%	57.0%	66.2%
**6**	79.7%	73.7%	89.4%	84.3%	84.2%	82.3%
**5**	77.0%	76.3%	91.1%	88.1%	72.6%	81.0%
**4**	77.0%	77.8%	86.7%	86.7%	75.6%	80.7%
**3**	82.2%	75.6%	85.2%	80.7%	79.3%	80.6%
**2**	83.7%	74.1%	86.7%	86.7%	78.5%	81.9%
**1**	83.7%	75.6%	90.4%	86.7%	80.7%	83.4%
**−1**	82.2%	68.9%	85.9%	85.9%	84.4%	81.5%
**−2**	80.0%	77.0%	82.2%	85.9%	79.3%	80.9%
**−3**	77.0%	72.6%	85.2%	89.6%	78.5%	80.6%
**−4**	75.6%	74.8%	89.6%	85.9%	78.5%	80.9%
**−5**	85.2%	80.0%	87.4%	87.4%	74.8%	83.0%
**−6**	81.5%	76.3%	88.1%	88.9%	79.3%	82.8%

**Table 5:** Mixed adjustment data test results.

Model	Angry	Fearful	Happy	Calm	Sad	Average
**Original**	76.3%	68.9%	73.3%	55.6%	57.0%	66.2%
**R40%&Pre**	85.2%	79.3%	96.3%	91.1%	79.3%	86.2%
**R40%&T—10%&Pre**	83.7%	77.8%	93.3%	93.3%	83.0%	86.2%
**T—10%&Pre**	90.3%	79.4%	95.3%	92.2%	77.8%	87.0%
**R40%&T—10%**	93.3%	82.2%	97.8%	93.3%	77.8%	88.9%

**Table 6:** The confusion matrix of the best sound adjustment model.

		Predicted				
		Happy	Sad	Angry	Calm	Fearful
**Actual**	**Happy**	93.3%	0.0%	4.4%	2.2%	0.0%
	**Sad**	0.0%	82.2%	0.0%	0.0%	17.8%
	**Angry**	2.2%	0.0%	97.8%	0.0%	0.0%
	**Calm**	0.0%	0.0%	0.0%	93.3%	6.7%
	**Fearful**	0.0%	20.0%	0.0%	2.2%	77.8%

**Table 7:** Comparison of accuracy between KNN, GoogLeNet, and the original method of this research.

Method	Training Time	Accuracy (Training)	Accuracy (Validation)	Accuracy (Testing)
**KNN**	1.5 (sec)	81.1%	-	71.2%
**GoogLeNet**	13.8 (min)	-	65.1%	51.2%
**DNN**	25.4 (sec)	93.3%	72.8%	66.2%

**Table 8:** Comparison of accuracy between KNN, GoogLeNet, and the proposed approach with 40% pre-emphasis.

Method	Training Time	Accuracy (Training)	Accuracy (Validation)	Accuracy (Testing)
**KNN**	5.4 (sec)	82.5%	-	76.6%
**GoogLeNet**	43.5 (min)	-	75.6%	66.5%
**DNN**	32.7 (sec)	95.2%	88.1%	86.2%

**Table 9:** Comparison of accuracy between KNN, GoogLeNet, and the proposed approach with rotation 40% and FM-10%.

Method	Training Time	Accuracy (Training)	Accuracy (Validation)	Accuracy (Testing)
**KNN**	5.3 (sec)	82.2%	-	75.7%
**GoogLeNet**	41.2 (min)	-	72.4%	66.7%
**DNN**	64.9 (sec)	97.0%	92.7%	88.9%

**Table 10:** Comparison of accuracy between KNN, GoogLeNet, and the proposed approach with rotation 40%, FM-10%, and pre-emphasis.

Method	Training Time	Accuracy (Training)	Accuracy (Validation)	Accuracy (Testing)
**KNN**	9.7 (sec)	84.1%	-	77.9%
**GoogLeNet**	56.8 (min)	-	81.0%	68.7%
**DNN**	50.9 (sec)	94.4%	89.1%	86.2%
